# Inpatient Capacity at Children’s Hospitals during Pandemic (H1N1) 2009 Outbreak, United States

**DOI:** 10.3201/eid1709.101950

**Published:** 2011-09

**Authors:** Marion R. Sills, Matthew Hall, Evan S. Fieldston, Paul D. Hain, Harold K. Simon, Thomas V. Brogan, Daniel B. Fagbuyi, Michael B. Mundorff, Samir S. Shah

**Affiliations:** Author affiliations: University of Colorado School of Medicine, Aurora, Colorado, USA (M.R. Sills);; Children's Hospital Colorado, Aurora (M.R. Sills);; Child Health Corporation of America, Shawnee Mission, Kansas, USA (M. Hall);; University of Pennsylvania School of Medicine, Philadelphia, Pennsylvania, USA (E.S. Fieldston, S.S. Shah); Children's Hospital of Philadelphia, Philadelphia (E.S. Fieldston, S.S. Shah);; Monroe Carell Jr. Children's Hospital at Vanderbilt, Nashville, Tennessee, USA (P.D. Hain);; Emory University School of Medicine, Atlanta, Georgia, USA (H.K. Simon);; Children's Healthcare of Atlanta, Atlanta (H.K. Simon); Seattle Children’s Hospital, Seattle, Washington, USA (T.V. Brogan);; University of Washington School of Medicine, Seattle (T.V. Brogan);; The George Washington University School of Medicine, Washington, DC, USA (D.B. Fagbuyi);; Children's National Medical Center, Washington (D.B. Fagbuyi);; Intermountain Healthcare, Salt Lake City, Utah, USA (M.B. Mundorff)

**Keywords:** hospital bed capacity, influenza A virus, (H1N1) subtype, pandemic (H1N1) 2009, child, pandemic, United States, viruses, research

## MEDSCAPE CME

Medscape, LLC is pleased to provide online continuing medical education (CME) for this journal article, allowing clinicians the opportunity to earn CME credit.

This activity has been planned and implemented in accordance with the Essential Areas and policies of the Accreditation Council for Continuing Medical Education through the joint sponsorship of Medscape, LLC and Emerging Infectious Diseases. Medscape, LLC is accredited by the ACCME to provide continuing medical education for physicians.

Medscape, LLC designates this Journal-based CME activity for a maximum of 1 *AMA PRA Category 1 Credit(s)*^TM^. Physicians should claim only the credit commensurate with the extent of their participation in the activity.

All other clinicians completing this activity will be issued a certificate of participation. To participate in this journal CME activity: (1) review the learning objectives and author disclosures; (2) study the education content; (3) take the post-test with a 70% minimum passing score and complete the evaluation at www.medscape.org/journal/eid; (4) view/print certificate.

**Release date: August 23, 2011; Expiration date: August 23, 2012**

## Learning Objectives

Upon completion of this activity, participants will be able to:

- Compare the 2009 H1N1 influenza pandemic with past influenza pandemics
- Evaluate the occupancy of children’s hospitals in the United States during the 2009 H1N1 influenza pandemic
- Analyze the relative effects of the 2009 H1N1 influenza pandemic on emergency departments and inpatient services
- Distinguish the number of additional admissions required in 2009 to push the children’s hospital system to full capacity.

## MEDSCAPE CME EDITOR

**Karen Foster, **Technical Writer/Editor, *Emerging Infectious Diseases. Disclosure: Karen Foster has disclosed no relevant financial relationships.*

## MEDSCAPE CME AUTHOR

**Charles P. Vega, MD**, Associate Professor; Residency Director, Department of Family Medicine, University of California, Irvine. *Disclosure: Charles P. Vega, MD, has disclosed no relevant financial relationships.*

## AUTHORS

*Disclosures:* ***Matthew Hall, PhD; Evan S. Fieldston, MD, MBA, MSHP; Paul D. Hain, MD; Thomas V. Brogan, MD;*** *and* ***Michael B. Mundorff, MBA, MHSA,*** *have disclosed no relevant financial relationships.* ***Marion R. Sills, MD, MPH,*** *has disclosed the following relevant financial relationships: received grants for clinical research from Agency for Healthcare Research and Quality.* ***Harold K. Simon, MD, MBA,*** *has disclosed the following relevant financial relationships: received grants for clinical research from Baxter International Inc. (rehydration clinical trial); AspenBio Pharma, Inc (appendicitis screening); National Institutes of Health (progesterone planning trial through the Pediatric Applied Research Network), all through Emory University.* ***Daniel B. Fagbuyi, MD,*** *has disclosed the following relevant financial relationships: owns stock, stock options, or bonds from Medco Health Solutions, Inc.; Orexigen Therapeutics, Inc.* ***Samir S. Shah, MD, MSCE,*** *has disclosed the following relevant financial relationships: received grants for clinical research from National Institutes of Health; Agency for Healthcare Research and Quality; Robert Wood Johnson Foundation.*
