# Supplementary material for: Inpatient Capacity at Children’s Hospitals during Pandemic (H1N1) 2009 Outbreak, United States
Source: Emerg Infect Dis. 2011 Sep;17(9):1685–91. doi: 10.3201/eid1709.101950 (PMC3320222; doi:10.3201/eid1709.101950)
Supplement: Technical Appendix — Seasonal influenza epidemic comparison data. [file 10-1950-Techapp_2p.pdf]

Article DOI: http://dx.doi.org/10.3201/eid1709.101950

Inpatient Capacity at Children's Hospitals during Pandemic (H1N1) 2009 Outbreak, United States

Technical Appendix

At baseline (all of 2008), during the 2008-09 seasonal influenza period, and during pandemic (H1N1) 2009, non-ICU and ICU occupancy levels were high. Occupancy in non-ICU beds was higher during the pandemic period compared with the 2008-09 seasonal influenza epidemic for 2 of 34 hospitals for which comparison data were available (Technical Appendix Figure 1). Occupancy in ICU beds was higher during the pandemic period than during the 2008-09 seasonal influenza pandemic period for 4 of 34 hospitals (Technical Appendix Figure 2). Across the 34 hospitals, the median ratio of 2009 pandemic period occupancy to 2008-9 seasonal influenza occupancy was 0.92 (0.86–0.96) for non-ICU beds and 0.90 (0.78–0.99) for ICU beds.

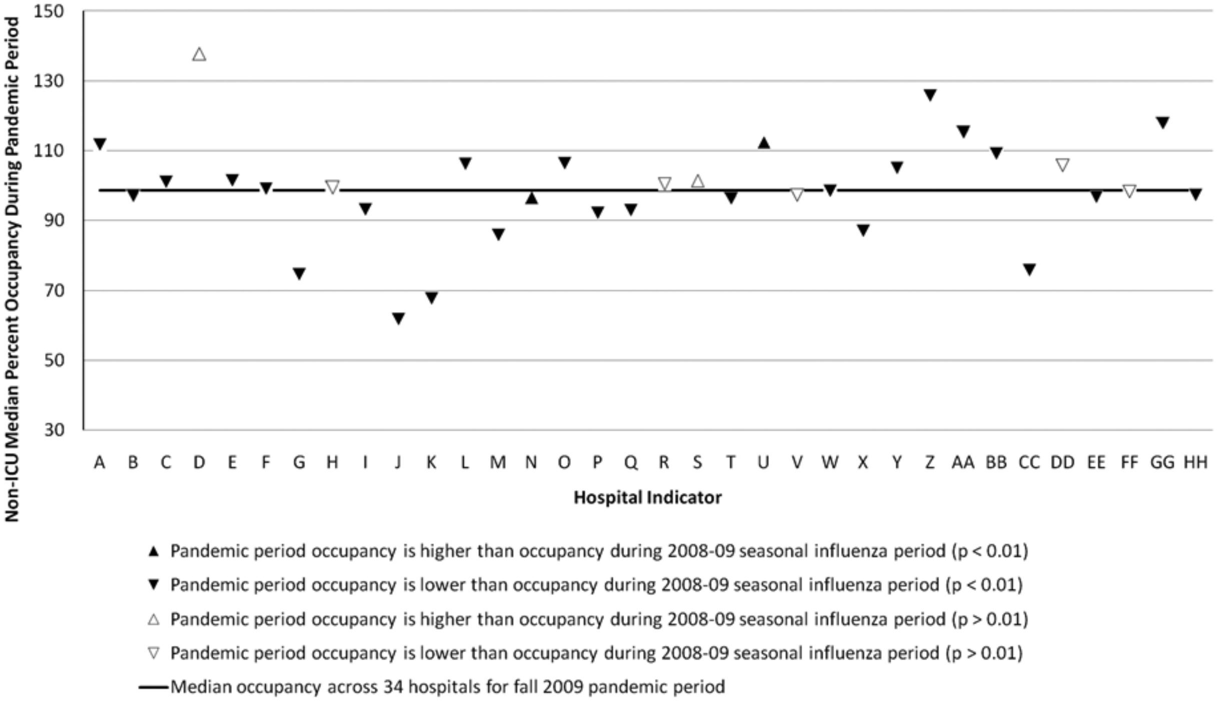

Technical Appendix Figure 1.

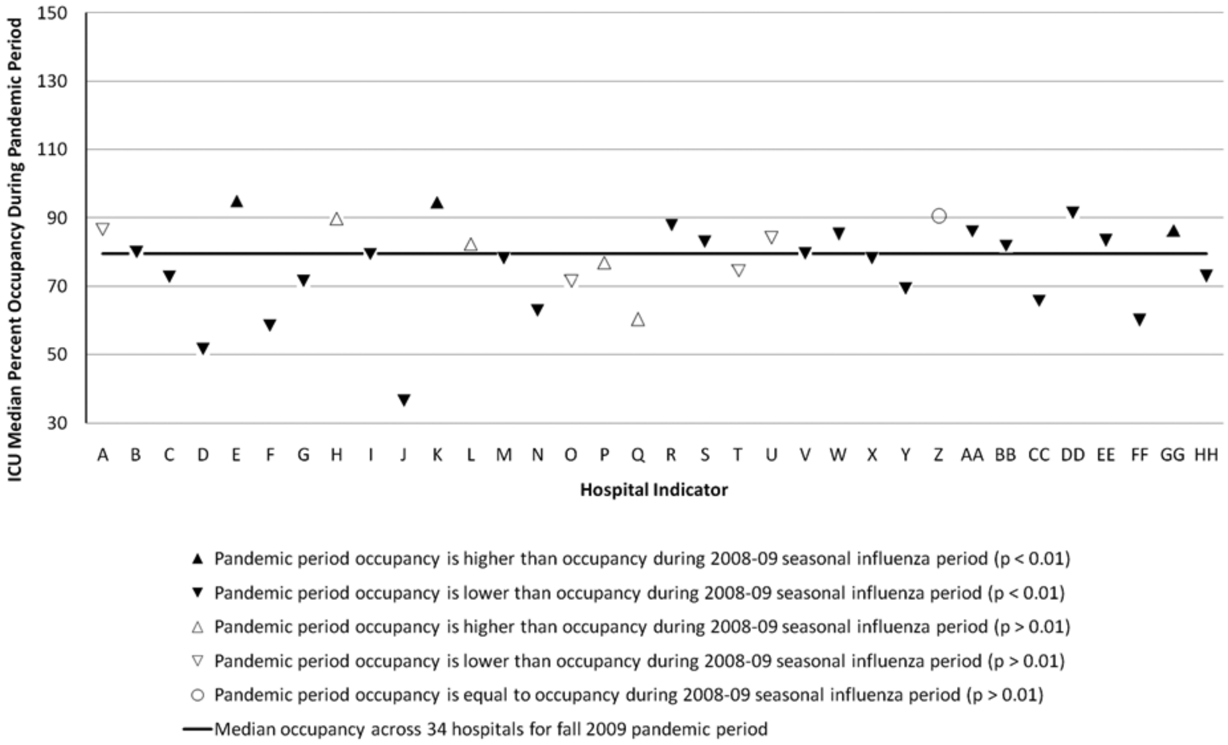

Technical Appendix Figure 2.
