# Supplementary material for: Inpatient Capacity at Children’s Hospitals during Pandemic (H1N1) 2009 Outbreak, United States
Source: Emerg Infect Dis. 2011 Sep;17(9):1685–91. doi: 10.3201/eid1709.101950 (PMC3320222; doi:10.3201/eid1709.101950)
Supplement: Related texts

# Research article: Inpatient Capacity at Children’s Hospitals during Pandemic (H1N1) 2009 Outbreak, United States

**DOI:** 10.3201/eid1709.101950

**Authors:** Marion R. Sills, Matthew Hall, Evan S. Fieldston, Paul D. Hain, Harold K. Simon, Thomas V. Brogan, Daniel B. Fagbuyi, Michael B. Mundorff, Samir S. Shah

**Affiliations:** Author affiliations: University of Colorado School of Medicine, Aurora, Colorado, USA (M.R. Sills);; Children's Hospital Colorado, Aurora (M.R. Sills);; Child Health Corporation of America, Shawnee Mission, Kansas, USA (M. Hall);; University of Pennsylvania School of Medicine, Philadelphia, Pennsylvania, USA (E.S. Fieldston, S.S. Shah); Children's Hospital of Philadelphia, Philadelphia (E.S. Fieldston, S.S. Shah);; Monroe Carell Jr. Children's Hospital at Vanderbilt, Nashville, Tennessee, USA (P.D. Hain);; Emory University School of Medicine, Atlanta, Georgia, USA (H.K. Simon);; Children's Healthcare of Atlanta, Atlanta (H.K. Simon); Seattle Children’s Hospital, Seattle, Washington, USA (T.V. Brogan);; University of Washington School of Medicine, Seattle (T.V. Brogan);; The George Washington University School of Medicine, Washington, DC, USA (D.B. Fagbuyi);; Children's National Medical Center, Washington (D.B. Fagbuyi);; Intermountain Healthcare, Salt Lake City, Utah, USA (M.B. Mundorff)

## Abstract

Quantifying how close hospitals came to exhausting capacity during the outbreak of pandemic influenza A (H1N1) 2009 can help the health care system plan for more virulent pandemics. This ecologic analysis used emergency department (ED) and inpatient data from 34 US children's hospitals. For the 11-week pandemic (H1N1) 2009 period during fall 2009, inpatient occupancy reached 95%, which was lower than the 101% occupancy during the 2008–09 seasonal influenza period. Fewer than 1 additional admission per 10 inpatient beds would have caused hospitals to reach 100% occupancy. Using parameters based on historical precedent, we built 5 models projecting inpatient occupancy, varying the ED visit numbers and admission rate for influenza-related ED visits. The 5 scenarios projected median occupancy as high as 132% of capacity. The pandemic did not exhaust inpatient bed capacity, but a more virulent pandemic has the potential to push children’s hospitals past their maximum inpatient capacity.

During March and April 2009, a novel influenza A (H1N1) virus began to spread in North America that disproportionately affected children, who constituted half of patients hospitalized for influenza-related illness (IRI) during spring 2009 (*1**–**3*). After a summertime decline, the virus returned to full activity in the fall, and children continued to have the highest rates of illness and hospitalization (*4*). As a result, pediatric providers and children’s hospitals cared for large numbers of patients with pandemic (H1N1) 2009 virus (*5**,**6*). Despite the high attack rate for children, the pandemic virus was milder than prior pandemic viruses. The attack rate for pandemic (H1N1) 2009 was lower for children (17.9%) (*7*) than it was for each of the past 3 pandemics in the United States (1918, 1958, and 1968) (21%–54%) (*8*), and the hospitalization rate was lower for children by >48-fold (0.17/1,000 symptomatic children (*9*) vs. estimates as high as 8.5/1,000 symptomatic children [10]).

Because of relatively low virulence, pandemic (H1N1) 2009 resulted in comparatively fewer hospitalizations than feared, but it greatly affected ambulatory settings and emergency departments (EDs) (*11**–**13*). The exact effect on children’s hospitals remains unknown because published studies have reported only regional data and have quantified hospital admissions rather than inpatient occupancy (*14**–**16*). Assessing use of capacity in the context of a low-virulence influenza pandemic can provide insight into how a more virulent virus might directly affect children’s hospitals and indirectly affect all health care systems throughout their catchment areas. Occupancy levels above and beyond existing capacity limits would represent a true crisis that would dramatically affect the already-stretched health care–delivery system (*17*). Because children’s hospitals play an integral role in coordinating health delivery (*18*), defining the limits of capacity reserve and quantifying how close these hospitals came to exhausting these limits can help the entire health care system better plan for more virulent pandemics or other disaster-type events.

With the effect of pandemic (H1N1) 2009 on children’s hospitals as a collective case study, we evaluated how close to full capacity US children’s hospitals functioned during the outbreak of pandemic (H1N1) 2009 and the implications for the health care systems had we not been fortunate regarding the low virulence of subtype H1N1 influenza (*19*). The objectives of this study were to 1) compare occupancy at US tertiary care children’s hospitals during the pandemic period with occupancy during the 2008–09 seasonal influenza outbreak, 2) measure how close each hospital came to exhausting capacity for inpatient beds, and 3) measure the effect on capacity if pandemic (H1N1) 2009 during fall 2009 had been more severe.

## Methods

### Source Data

This ecologic analysis used data from the Pediatric Health Information System (PHIS), which includes ED and inpatient data from 41 free-standing nonprofit tertiary care children’s hospitals in all regions of the United States. The Child Health Corporation of America (Shawnee Mission, KS, USA) and participating hospitals jointly validate data quality and reliability (*20*). This analysis comprises data from the 34 PHIS hospitals that provided codes indicating intensive care unit (ICU) and non-ICU bed designations for the study period.

### Study Participants

We defined the pandemic (H1N1) 2009 period and other influenza epidemic periods using national influenza circulation data obtained from the World Health Organization collaborating laboratories and the National Respiratory and Enteric Virus Surveillance System (*21*). Using as a threshold the weeks with >20% positive test results as reported in the Morbidity and Mortality Weekly Report (Centers for Disease Control and Prevention, Atlanta, GA, USA) (*22**,**23*), we defined the period of pandemic (H1N1) 2009 as September 5–November 20, 2009. To compare inpatient resource use during this period with that during a seasonal influenza period, we used the weeks of seasonal influenza from the 2008–09 season (January 31–March 20, 2009), defined using the same 20% threshold (*23*).

Because specifically identifying patients with pandemic (H1N1) 2009 was not feasible, we used a standard list of International Classification of Diseases, Ninth Revision codes developed for measuring IRI to determine resource use of inpatient beds (*5*). This list comprises International Classification of Diseases, 9th Revision, codes 460–496 or 510–519 as the primary or secondary discharge diagnosis and captures not only primary infections with influenza, but also secondary infections (e.g., bacterial pneumonia) and exacerbations of other conditions (e.g., asthma).

### Measures

Our primary measure was midnight occupancy for non-ICU beds and for ICU beds. The numerator for occupancy comprised all children (age 0–18 y; median age 3.1 y, interquartile range [IQR] 1.0–8.1 y) occupying non-ICU and ICU beds on each day of the study period. We obtained denominator data (i.e., annual number of licensed, in-service beds) from the Child Health Corporation of America and confirmed them by an email survey to each hospital’s designated PHIS contact. Step-down beds were categorized as non-ICU. If a patient spent at least 1 midnight in an ICU bed during his or her hospital stay, admission was considered an ICU admission and was not counted as a non-ICU admission. We included all hospitalized patients of any admission status (observation or inpatient) to fully quantify hospital occupancy. We excluded newborn nursery and mental health admissions and those designated beds from the analysis.

For our second objective, we defined the threshold as 100% occupancy on the basis of licensed, in-serviced beds as capacity. Although lower thresholds have been suggested as the point at which quality and safety decline (*24**,**25*), 100% represents the scenario in which a hospital has actually exhausted its typical capacity of in-service beds. In calculating non-ICU and ICU occupancy, we counted the number of patients in each bed type at the midnight at the end of the day.

For our third objective, we analyzed the 26 PHIS hospitals for which ED data were available. In our models, we varied 2 parameters and described the effect on inpatient occupancy: 1) number of ED IRI visits and 2) ED-to-hospital admission rate. For the first, we used estimates from the 2 most recent, severe prior influenza pandemics (1957 and 1968), when the estimated upper bound of the attack rate was 36% (*9**,**10*). Estimates of the attack rate for pandemic (H1N1) 2009 for April–December 2009 were 17.9%, based on 55 million cases (*7*) in a July 2009 population of 307 million (*26*). Assuming the per-case rate of ED visits remained fixed, we estimated that ED IRI visits could have been 2× what they were if the attack rate had been similar to these 2 prior pandemics.

For the second parameter, we used the ED admission rate of 14.0% observed during the 2003–04 seasonal influenza weeks (November 1, 2003–January 9, 2004), 1 of the most severe recent influenza seasons (*27**,**28*). We also modeled a 30% admission rate (the upper end of overall ED admission rate for study hospitals in 2008), which actually falls well below the rate projected from hospitalization estimates of earlier influenza pandemics and epidemics (*9**,**10*).

### Analysis

To compare occupancy during the fall pandemic (H1N1) 2009 period with baseline, we calculated the number of admissions, bed-days, and the occupancy for all beds, non-ICU beds, and ICU beds for the 2009 pandemic period and for 2 comparison periods: the entire prior calendar year (2008) and the prior seasonal influenza period. We assessed the statistical significance of the difference in median occupancy between the 2009 pandemic period and the seasonal influenza comparison period using the Wilcoxon rank-sum test.

To measure how close each hospital came to exhausting capacity, we calculated how many additional non-ICU and ICU patients could have been admitted by each hospital. For each day, we counted the number of unoccupied beds of each type and modeled how many additional patients were needed to fill all available beds for each hospital. For hypothetical additional patients, we modeled patients’ continued presence iteratively for each day of the study period as non-ICU and ICU patients on the basis of the characteristics of patients admitted during the fall 2009 pandemic with IRI (e.g., 20% with a 1-day length of stay, 35% with a 2-day stay, 30% with a 3-day stay). For both models, we assigned bed-days of each stay to each respective area, ICU and non-ICU. To index the total number of additional patients needed to fill the hospital to capacity across hospitals, we then calculated the number of additional patients per 10 beds (non-ICU or ICU).

To measure the effect on capacity of a more severe outbreak of pandemic (H1N1) 2009, we calculated the number of ED IRI visits and the ED-to-inpatient admission rate for ED IRI visits for the 26 PHIS hospitals for which ED data were available. We then used the same modeling methods described above to model the number of additional bed-days (non-ICU, ICU) in each scenario. We expressed findings from the 6 scenarios in 2 ways: 1) percentage of hospital days >100% and 2) as median (IQR) occupancy. In these models, we made 4 assumptions. First, we assumed that the rate of non-IRI ED admissions remained unchanged. Second, we assumed that hospitals did not react to high occupancy by rescheduling elective admissions, an assumption based on a prior analysis of the same data set (*17*). Third we assumed that the number of ICU and non-ICU beds remained fixed for each calendar year. Fourth, we assumed that the inpatient length-of-stay distribution was not shifted toward longer hospitalizations during a more virulent pandemic.

We performed all analyses with SAS version 9.2 (SAS Institute, Inc., Cary, NC, USA) and considered p values <0.05 statistically significant. The study protocol was approved by the Colorado Multiple Institutional Review Board with a waiver of informed consent.

## Results

The 11-week period of evaluation during the fall 2009 pandemic period included a median of 2,774 (IQR 2,219–3,319) admissions and 19,283 (IQR 15,842–21,315) bed-days (Table 1). Median overall inpatient occupancy was 95% (IQR 85%–99%), whereas median overall occupancy during the 2008–09 seasonal influenza period was 101% (IQR 96%–110%) and, for the entire calendar year 2008, 91% (IQR 87%–95%). Hospitals’ experiences varied considerably, with hospital-level median occupancy ranging from 57.4% to 128.0% (Technical Appendix). To reach 100% occupancy during the pandemic period, for every 10 beds of each type, hospitals would have needed to admit a median of 0.2 (IQR 0.1–0.3) additional patients per day for non-ICU beds and 0.7 (IQR 0.5–0.9) per day for ICU beds (Table 2).

**Table 1:** Hospital characteristics and aggregated experiences in 34 PHIS hospitals, United States, all of 2008, 2008–09 seasonal influenza period, and fall 2009 pandemic (H1N1) 2009 period*

Hospital characteristics	Calendar year 2008, 52 wk, median (IQR)	2008–09 seasonal influenza, 8 wk, median (IQR)†	Fall 2009 pandemic influenza, 11 wk, median (IQR)‡
Overall§			
Beds, no.	255 (221–316)	265 (219–319)	265 (219–319)
Admissions, no.	12,105 (9,913–14,247)	1,871 (1,489–2,197)	2,774 (2,219–3,319)
Bed-days, no.	89,117 (70,691–96,346)	15,299 (13,087–17,312)	19,283 (15,842–21,315)
Occupancy, %	91 (87–95)	101 (96–110)	95 (85–99)
Non-ICU			
Beds, no.	129 (91–179)	132 (90–181)	132 (90–181)
Admissions, no.	10,575 (8,415–13,022)	1,595 (1,346–2,030)	2,419 (1,838–2,907)
Bed-days, no.	81,735 (65,703–87,171)	13,933 (11,946–15,902)	16,703 (1,2051–18,843)
Occupancy, %	94 (91–103)	108 (100–117)	98 (92–105)
ICU¶			
Beds, no.	33 (25–48)	37 (27–51)	37 (27–51)
Admissions, no.	1,654 (1,167–22,80)	245 (189–316)	401 (296–516)
Bed-days, no.	8,299 (5,502–11,083)	1,576 (1,155–1,889)	1,873 (1,094–2,638)
Occupancy, %	72 (44–87)	80 (51–97)	65 (47–88)

*PHIS, Pediatric Health Information System; IQR, interquartile range; ICU, intensive care unit; MMWR, Morbidity and Mortality Weekly Report (Centers for Disease Control and Prevention, Atlanta, GA, USA). †MMWR reporting weeks 4–11 (January 31–March 20, 2009). ‡MMWR reporting weeks 35–45 (September 5–November 20, 2009). §Excluding neonatal and behavioral health patients and designated beds. ¶Excluding neonatal intensive care units.

**Table 2:** Additional admissions needed to fill hospitals to 100% occupancy during 11-week fall 2009 pandemic (H1N1) 2009 period, per 10 beds per day, in study of 34 PHIS hospitals, United States*

Measure	Non-ICU	ICU
Minimum	0.0	0.2
1st quartile	0.1	0.5
Median†	0.2	0.7
3rd quartile	0.3	0.9
Maximum	1.3	2.0

*PHIS, Pediatric Health Information System; ICU, intensive care unit. †Median indicates the hospital requiring the median number of patients to fill all beds during the pandemic period.

For the 26 hospitals for which ED and inpatient data were available, the median ED-to-hospital admission rate for IRI patients was 5.4% (IQR 3.3%–8.1%). Different hypothetical scenarios for ED IRI volume and admission rates would have differently affected the frequency of hospital days exceeding the 100% occupancy threshold for exhausting capacity reserves (Table 3). The actual experience in 2009 (scenario A) resulted in 23.3% of hospital days with >100% occupancy across the 26 hospitals. Had the hospitals instead experienced the IRI admit rate from the 2003–04 influenza season (14.0%) applied to the same volume of patients, 37.6% of hospital days would have been >100% full (scenario B). Had the admission rate been 30% for 2× the volume of patients, 85.7% of hospital days would have been >100% full, exhausting capacity reserves (scenario F).

**Table 3:** Predictive model of the percentage of hospital days (ICU and non-ICU) during at >100% hospital occupancy, using 11-week pandemic period fall 2009 pandemic period data as baseline occupancy, in study of 34 PHIS hospitals, United States*

ED IRI volume	ED IRI admission rate		
	Fall 2009 pandemic period†	2003–04 seasonal influenza period‡	Hypothetical
Admission rate, %	5.4	14.0	30
Same as during fall 2009 pandemic period, median (IQR)†	A: 23.3 (0–42.9)	B: 37.6 (11.6–58.4)	C: 63.6 (23.4–83.1)
100% increase from pandemic period, median (IQR)	D: 64.9 (16.9–81.8)	E: 72.7 (24.6–81.8)	F: 85.7 (40.2–98.7)

*ICU, intensive care unit; PHIS, Pediatric Health Information System; ED, emergency department; IRI, influenza-related illness; MMWR, Morbidity and Mortality Weekly Report (Centers for Disease Control and Prevention, Atlanta, GA, USA). Letters correspond to scenarios shown in the Figure. †MMWR reporting weeks 35–45 (September 5–November 20, 2009). ‡MMWR reporting weeks 44–53 (November 1, 2003–January 9, 2004).

Individual hospital experience varied considerably (Figure). For each hospital, the dot-plots we constructed show the distribution of occupancy data across hospitals for each of the 6 scenarios. For our worst-case scenario (scenario F), median occupancy would have been 132% (IQR 124%–145%).

**Figure:** Predictive model of hospital occupancy during 11-week outbreak of pandemic influenza A (H1N1) 2009 in the United States, by ED IRI admission rate and ED IRI volume, using fall 2009 pandemic period data as baseline. Percentages given indicate hospital admission rate during period or for hypothetical scenario. Gray area indicates 100% occupancy. Each circle represents median occupancy from 1 hospital; vertical whiskers indicate interquartile range. y-axes indicate percentage occupancy; x-axes indicates individual hospitals. Median (interquartile range) occupancy across study hospitals: A) 95.9% (85.0%–100.3%); B) 97.9% (80.5%–102.7%); C) 109.7% (97.9%–117.1%); D) 102.2% (91.7%–112.4%); E) 108.0% (96.4%–114.9%); F) 132.4% (124.5%–145.5%). Letters correspond to scenarios in Table 3. *MMWR reporting weeks 35–45 (September 5–November 20, 2009). †MMWR reporting weeks 44–53 (November 1, 2003–January 9, 2004). ED, emergency department; IRI, influenza-related illness; MMWR, Morbidity and Mortality Weekly Report (Centers for Disease Control and Prevention, Atlanta, GA, USA).

## Discussion

We examined the effect on children’s hospitals’ resources during fall 2009 when pandemic influenza A (H1N1) 2009 virus was active. We demonstrated that children’s hospitals faced high levels of occupancy (median 95%) in regular inpatient care areas and ICUs, but this situation did not differ from typical levels of high occupancy commonly experienced at some hospitals. Despite the mild virulence of pandemic (H1N1) 2009 virus, children’s hospitals needed only <1 additional admission per 10 inpatient beds to reach 100% occupancy. Additionally, the pandemic occurred during early fall, when viruses that cause respiratory and gastrointestinal illnesses (which typically increase occupancy at children’s hospitals) were not circulating widely. Models representing an outbreak of a more virulent influenza virus based on historical comparisons demonstrate that modest increases in ED visits or ED admission rates would have resulted in substantial overcrowding among the large cohort of children’s hospitals in our study.

These findings are notable in the context of national disaster planning related to children. The National Commission on Children and Disasters’ 2010 Report to the President and Congress recommended that additional resources provide a “formal regionalized pediatric system of care to support pediatric surge capacity” and emphasizes that children’s hospitals are central to such regionalization (*18*). Our study shows that children’s hospitals, the central component of this proposed regionalized system, routinely operate so close to capacity that little available reserve exists for even a modest surge of inpatients. For a hospital with 150 non-ICU beds and 50 ICU beds, an additional 3.0 non-ICU and 3.5 ICU admissions per day would have exhausted capacity. Although the 2009 influenza pandemic did not do so, surge capacity is scarce, as demonstrated by the many hospitals that are already operating at or near maximum capacity in their EDs and inpatient areas (*17**,**29*).

Federal planners have suggested that surge capacity should accommodate 500 inpatients per million population, but such capacity does not exist for children under normal circumstances; capacity for only 193 inpatients per million children is available during typical winter weekdays (*29**–**31*). Although we expressed our findings in terms of hospital occupancy rather than on a population basis, our findings are similar to those raising alarm about limited inpatient capacity in the face of a pandemic or disaster.

Pandemics extend over many weeks and affect large regions, if not the entire country. Although the hospitals may be able to handle such levels of occupancy on a short-term basis, whether they could do so for prolonged periods is unclear. Even though a health care system’s capacity reserve cannot be designed on a daily basis to handle a pandemic, the frequent level of high occupancy already experienced by children’s hospitals and the resulting lack of a buffer for a pandemic-associated surge should be considered by individuals and organizations involved with planning and disaster preparedness (*32**,**33*). Planning for such events at hospital and regional levels may be improved with data about current capacity reserves and how perturbations can affect that capacity.

In previous studies of large-scale epidemics, hospitals have altered standards of care—as occurred in Toronto during the 2003 outbreak of severe acute respiratory syndrome—to meet increased patient needs (*30**,**34**,**35*). During the outbreak of severe acute respiratory syndrome, restrictions on scheduled (i.e., elective) admissions were imposed in Toronto (*36*). Although we did not study scheduled admissions, our analysis suggests in a more virulent pandemic (scenario F), hospitals would have run out of space even if they had rescheduled the 15%–25% of scheduled pediatric admissions; this percentage includes the 20% of elective admissions for chemotherapy, a treatment that is not amenable to prolonged postponement (*37*).

Our findings are subject to several limitations. The 34 hospitals in this study represent a subset of the ≈250 US children's hospitals and may not be representative of these children’s hospitals or of other hospitals that admit children, even though the study included children’s hospitals in all regions of the country. The analysis did not consider measures that individual hospitals and regional systems might use to reduce occupancy, such as canceling scheduled admissions, which would have caused us to overestimate occupancy. On the other hand, our assumption about length of stay would have caused us to underestimate occupancy. Our analysis used midnight census; actual daytime occupancy most likely was higher (*38*), and thus true surge capacity was even lower than estimated. Finally, the modeled scenarios were based on historical comparisons, which represent a range of potential demands on the health care system.

For hospitals and government agencies, the results of our study should prompt review of preparedness planning and reconsideration of surge capacity. Systemwide resource limitations must be considered because ambulatory and inpatient services interrelate. The outbreak of low-virulence pandemic (H1N1) 2009 virus affected EDs disproportionately but left inpatient services relatively unaffected (*13*). Exploring the parameters of more severe epidemics might allow planners at individual hospitals, as well as regional health administrators, to consider what alterations in standards may be necessary.

## References

[1] Centers for Disease Control and Prevention. Update: novel influenza A (H1N1) virus infection—Mexico, March–May 2009. MMWR Morb Mortal Wkly Rep. 2009;58:585–9.19498336PMC5856103

[2] Thompson WW, Shay DK, Weintraub E, Brammer L, Bridges CB, Cox NJ, Influenza-associated hospitalizations in the United States. JAMA. 2004;292:1333–40. 10.1001/jama.292.11.133315367555

[3] Jain S, Kamimoto L, Bramley AM, Schmitz AM, Benoit SR, Louie J, Hospitalized patients with 2009 H1N1 influenza in the United States, April–June 2009. N Engl J Med. 2009;361:1935–44. 10.1056/NEJMoa090669519815859

[4] Reed C, Angulo FJ, Swerdlow DL, Lipsitch M, Meltzer MI, Jernigan D, Estimates of the prevalence of pandemic (H1N1) 2009, United States, April–July 2009. Emerg Infect Dis. 2009;15:2004–7. 10.3201/eid1512.09141319961687PMC3375879

[5] Izurieta HS, Thompson WW, Kramarz P, Shay DK, Davis RL, DeStefano F, Influenza and the rates of hospitalization for respiratory disease among infants and young children. N Engl J Med. 2000;342:232–9. 10.1056/NEJM20000127342040210648764

[6] O’Brien MA, Uyeki TM, Shay DK, Thompson WW, Kleinman K, McAdam A, Incidence of outpatient visits and hospitalizations related to influenza in infants and young children. Pediatrics. 2004;113:585–93. 10.1542/peds.113.3.58514993554

[7] Centers for Disease Control and Prevention. Updated CDC estimates of 2009 H1N1 influenza cases, hospitalizations and deaths in the United States, April 2009–April 10, 2010 [cited 2010 Jul 21]. http://www.flu.gov/individualfamily/about/h1n1/estimates_2009_h1n1.html

[8] Glezen WP. Emerging infections: pandemic influenza. Epidemiol Rev. 1996;18:64–76.887733110.1093/oxfordjournals.epirev.a017917

[9] Presanis AM, De Angelis D, New York City Swine Flu Investigation Team, Hagy A, Reed C, Riley S, . The severity of pandemic H1N1 influenza in the United States, from April to July 2009: a Bayesian analysis. PLoS Med. 2009;6:e1000207. 10.1371/journal.pmed.100020719997612PMC2784967

[10] Meltzer MI, Cox NJ, Fukuda K. The economic impact of pandemic influenza in the United States: priorities for intervention. Emerg Infect Dis. 1999;5:659–71. 10.3201/eid0505.99050710511522PMC2627723

[11] Costello BE, Simon HK, Massey R, Hirsh DA. Pandemic H1N1 influenza in the pediatric emergency department: a comparison with previous seasonal influenza outbreaks. Ann Emerg Med. 2010;56:643–8. 10.1016/j.annemergmed.2010.03.00120363533

[12] Miroballi Y, Baird JS, Zackai S, Cannon JM, Messina M, Ravindranath T, Novel influenza A(H1N1) in a pediatric health care facility in New York City during the first wave of the 2009 pandemic. Arch Pediatr Adolesc Med. 2010;164:24–30. 10.1001/archpediatrics.2009.25920048238

[13] Sills MR, Hall M, Simon HK, Fieldston ES, Walter N, Levin JE, Resource burden at children's hospitals experiencing surge volumes during the spring 2009 H1N1 influenza pandemic. Acad Emerg Med. 2011;18:158–66. 10.1111/j.1553-2712.2010.00992.x21314775

[14] Centers for Disease Control and Prevention. 2009 pandemic influenza A (H1N1) virus infections—Chicago, Illinois, April–July 2009. MMWR Morb Mortal Wkly Rep. 2009;58:913–8.19713879

[15] Ginocchio CC, Zhang F, Manji R, Arora S, Bornfreund M, Falk L, Evaluation of multiple test methods for the detection of the novel 2009 influenza A (H1N1) during the New York City outbreak. J Clin Virol. 2009;45:191–5. 10.1016/j.jcv.2009.06.00519540158PMC7172175

[16] Louie JK, Acosta M, Winter K, Jean C, Gavali S, Schechter R, Factors associated with death or hospitalization due to pandemic 2009 influenza A(H1N1) infection in California. JAMA. 2009;302:1896–902. 10.1001/jama.2009.158319887665

[17] Fieldston ES, Hall M, Sills MR, Slonim AD, Myers AL, Cannon C, Children's hospitals do not acutely respond to high occupancy. Pediatrics. 2010;125:974–81. 10.1542/peds.2009-162720403931PMC2913552

[18] National Commission on Children and Disasters. 2010 report to the President and Congress. AHRQ publication no. 10-M037. Rockville (MD): Agency for Healthcare Research and Quality. October 2010 [cited 2011 Jul 18]. http://www.ahrq.gov/prep/nccdreport

[19] Chan M. Time to get back on track to meet the Millennium Development Goals: address to Sixty-Third World Health Assembly. 2010 May 17 [cited 2010 Aug 14]. http://www.who.int/dg/speeches/2010/WHA_address_20100517/en/index.html

[20] Fletcher DM. Achieving data quality. how data from a pediatric health information system earns the trust of its users. J AHIMA. 2004;75:22–6.15559835

[21] Centers for Disease Control and Prevention. 2009 H1N1 flu: situation update [cited 2009 Sep 16]. http://www.cdc.gov/flu/weekly/fluactivity.htm#OIS

[22] Centers for Disease Control and Prevention. Influenza viruses isolated by WHO/NREVSS collaborating laboratories, 2009–2010 season [cited 2011 Jul 19]. http://www.cdc.gov/flu/weekly/weeklyarchives2009-2010/data/whoAllregt20.htm

[23] Centers for Disease Control and Prevention. Influenza viruses isolated by WHO/NREVSS collaborating laboratories 2008–2009 season [cited 2011 Jul 19]. http://www.cdc.gov/flu/weekly/weeklyarchives2008-2009/data/whoAllregt39.htm

[24] Hillier DF, Parry GJ, Shannon MW, Stack AM. The effect of hospital bed occupancy on throughput in the pediatric emergency department. Ann Emerg Med. 2009;53:767–76.e3.10.1016/j.annemergmed.2008.11.02419254815

[25] DeLia D. Hospital capacity, patient flow, and emergency department use in New Jersey. New Brunswick (NJ): Rutgers Center for State Health Policy; 2007 [cited 2011 Jul 18]. http://www.state.nj.us/health/rhc/documents/ed_report.pdf

[26] US Census Bureau. Annual population estimates 2000 to 2009 [cited 2010 Jul 21]. http://www.census.gov/popest/states/NST-ann-est.html

[27] Centers for Disease Control and Prevention. Update: influenza activity—United States and worldwide, 2003–04 season, and composition of the 2004–05 influenza vaccine. MMWR Morb Mortal Wkly Rep. 2010;53:547–52.15229411

[28] Centers for Disease Control and Prevention. Estimates of deaths associated with seasonal influenza—United States, 1976–2007. MMWR Morb Mortal Wkly Rep. 2010;59:1057–62.20798667

[29] Kanter RK. Pediatric mass critical care in a pandemic. Pediatr Crit Care Med. 2010 Oct 28; [Epub ahead of print].10.1097/PCC.0b013e3181fe390a21037504

[30] Kanter RK, Moran JR. Pediatric hospital and intensive care unit capacity in regional disasters: expanding capacity by altering standards of care. Pediatrics. 2007;119:94–100. 10.1542/peds.2006-158617200275

[31] Kanter RK, Moran JR. Hospital emergency surge capacity: an empiric New York statewide study. Ann Emerg Med. 2007;50:314–9. 10.1016/j.annemergmed.2006.10.01917178173

[32] Hick JL, O’Laughlin DT. Concept of operations for triage of mechanical ventilation in an epidemic. Acad Emerg Med. 2006;13:223–9. 10.1111/j.1553-2712.2006.tb01677.x16400088

[33] Cachon G, Terwiesch C. Matching supply with demand: an introduction to operations management. New York: McGraw-Hill; 2006.

[34] Schull MJ, Stukel TA, Vermeulen MJ, Guttmann A, Zwarenstein M. Surge capacity associated with restrictions on nonurgent hospital utilization and expected admissions during an influenza pandemic: lessons from the Toronto severe acute respiratory syndrome outbreak. Acad Emerg Med. 2006;13:1228–31. 10.1111/j.1553-2712.2006.tb01653.x16807399

[35] Institute of Medicine. Guidance for establishing standards of care for use in disaster situations. Washington: National Academies Press; 2009 [cited 2011 Jul 18]. http://books.nap.edu/openbook.php?record_id=1274925032361

[36] Schull MJ, Stukel TA, Vermeulen MJ, Zwarenstein M, Alter DA, Manuel DG, Effect of widespread restrictions on the use of hospital services during an outbreak of severe acute respiratory syndrome. CMAJ. 2007;176:1827–32. 10.1503/cmaj.06117417576979PMC1891122

[37] Ryan K, Levit K, Davis PH. Characteristics of weekday and weekend hospital admissions. Rockville (MD): Agency for Healthcare Research and Quality; 2010. Report no. 87 [cited 2011 Jul 18]. http://www.hcup-us.ahrq.gov/reports/statbriefs/sb87.pdf21452494

[38] DeLia D. Annual bed statistics give a misleading picture of hospital surge capacity. Ann Emerg Med. 2006;48:384–8. 10.1016/j.annemergmed.2006.01.02416997673

# Research article: Inpatient Capacity at Children’s Hospitals during Pandemic (H1N1) 2009 Outbreak, United States

**DOI:** 10.3201/eid1709.101950

**Authors:** Marion R. Sills, Matthew Hall, Evan S. Fieldston, Paul D. Hain, Harold K. Simon, Thomas V. Brogan, Daniel B. Fagbuyi, Michael B. Mundorff, Samir S. Shah

**Affiliations:** Author affiliations: University of Colorado School of Medicine, Aurora, Colorado, USA (M.R. Sills);; Children's Hospital Colorado, Aurora (M.R. Sills);; Child Health Corporation of America, Shawnee Mission, Kansas, USA (M. Hall);; University of Pennsylvania School of Medicine, Philadelphia, Pennsylvania, USA (E.S. Fieldston, S.S. Shah); Children's Hospital of Philadelphia, Philadelphia (E.S. Fieldston, S.S. Shah);; Monroe Carell Jr. Children's Hospital at Vanderbilt, Nashville, Tennessee, USA (P.D. Hain);; Emory University School of Medicine, Atlanta, Georgia, USA (H.K. Simon);; Children's Healthcare of Atlanta, Atlanta (H.K. Simon); Seattle Children’s Hospital, Seattle, Washington, USA (T.V. Brogan);; University of Washington School of Medicine, Seattle (T.V. Brogan);; The George Washington University School of Medicine, Washington, DC, USA (D.B. Fagbuyi);; Children's National Medical Center, Washington (D.B. Fagbuyi);; Intermountain Healthcare, Salt Lake City, Utah, USA (M.B. Mundorff)

## Earning CME Credit

To obtain credit, you should first read the journal article. After reading the article, you should be able to answer the following, related, multiple-choice questions. To complete the questions (with a minimum 70% passing score) and earn continuing medical education (CME) credit, please go to www.medscape.org/journal/eid. Credit cannot be obtained for tests completed on paper, although you may use the worksheet below to keep a record of your answers. You must be a registered user on Medscape.org. If you are not registered on Medscape.org, please click on the New Users: Free Registration link on the left hand side of the website to register. Only one answer is correct for each question. Once you successfully answer all post-test questions you will be able to view and/or print your certificate. For questions regarding the content of this activity, contact the accredited provider, CME@medscape.net. For technical assistance, contact CME@webmd.net. American Medical Association’s Physician’s Recognition Award (AMA PRA) credits are accepted in the US as evidence of participation in CME activities. For further information on this award, please refer to http://www.ama-assn.org/ama/pub/category/2922.html. The AMA has determined that physicians not licensed in the US who participate in this CME activity are eligible for *AMA PRA Category 1 Credits*™. Through agreements that the AMA has made with agencies in some countries, AMA PRA credit may be acceptable as evidence of participation in CME activities. If you are not licensed in the US, please complete the questions online, print the certificate and present it to your national medical association for review.

## Article Title: Inpatient Capacity at Children’s Hospitals during Pandemic (H1N1) 2009 Outbreak, United States

## CME Questions

**1. You sit on a planning commission for children's healthcare in your region, and the commission is reviewing health system performance during the 2009 H1N1 influenza pandemic. Overall, how did this pandemic compare with prior influenza pandemics among children in the United States?**A. H1N1 had a lower attack rate and a lower case-hospitalization rateB. H1N1 had a lower attack rate but a higher case-hospitalization rateC. H1N1 had a higher attack rate but a lower case-hospitalization rateD. H1N1 had a higher attack rate and a higher case-hospitalization rate**2. On the basis of the current study, what can you tell the commission in regard to the inpatient occupancy rate among children's hospitals during the 2009 H1N1 influenza pandemic?**A. It never exceeded 85%B. It was lower than that of the 2008-2009 influenza seasonC. It surged higher compared with occupancy rates immediately before and after the pandemicD. It could have accommodated 50% more admissions before going over 100% of capacity**3. What should your commission consider in regard to the virulence of influenza and hospital occupancy?**A. The 2009 H1N1 influenza pandemic affected inpatient occupancy more than emergency department capacityB. The 2009 H1N1 influenza pandemic affected inpatient occupancy and emergency department capacity equallyC. The emergency department-to-hospital admission rate for influenza-related illness patients was slightly more than 5% in 2009D. Higher acuity of influenza cases will probably have little effect on hospital occupancy rates**4. Approximately how many additional admissions per 10 hospital beds would have raised the overall hospital occupancy to 100% during the 2009 H1N1 influenza pandemic?**A. 1B. 4C. 7D. 9

## Activity Evaluation

**Table:** 

1. The activity supported the learning objectives.				
Strongly Disagree				Strongly Agree
1	2	3	4	5
2. The material was organized clearly for learning to occur.				
Strongly Disagree				Strongly Agree
1	2	3	4	5
3. The content learned from this activity will impact my practice.				
Strongly Disagree				Strongly Agree
1	2	3	4	5
4. The activity was presented objectively and free of commercial bias.				
Strongly Disagree				Strongly Agree
1	2	3	4	5

## Supplementary Material

Technical Appendix Seasonal influenza epidemic comparison data.
